# Local control and short-term outcomes after video-assisted thoracoscopic surgery segmentectomy versus lobectomy for pT1c pN0 non-small-cell lung cancer

**DOI:** 10.1093/icvts/ivad037

**Published:** 2023-02-14

**Authors:** Céline Forster, Etienne Abdelnour-Berchtold, Benoît Bédat, Jean Yannis Perentes, Matthieu Zellweger, Marc-Olivier Sauvain, Michel Christodoulou, Frédéric Triponez, Wolfram Karenovics, Thorsten Krueger, Michel Gonzalez

**Affiliations:** Department of Thoracic Surgery, Lausanne University Hospital (CHUV), Lausanne, Switzerland; Department of Thoracic Surgery, Lausanne University Hospital (CHUV), Lausanne, Switzerland; Department of Thoracic Surgery, University of Geneva (HUG), Geneva, Switzerland; Department of Thoracic Surgery, Lausanne University Hospital (CHUV), Lausanne, Switzerland; Faculty of Biology and Medicine, University of Lausanne (UNIL), Lausanne, Switzerland; Department of Thoracic Surgery, Lausanne University Hospital (CHUV), Lausanne, Switzerland; Department of General Surgery, Réseau Hospitalier Neuchâtelois (RHNe), Neuchâtel, Switzerland; Department of Thoracic Surgery, Réseau Santé du Valais Romand (RSVr), Sion, Switzerland; Department of Thoracic Surgery, University of Geneva (HUG), Geneva, Switzerland; Department of Thoracic Surgery, University of Geneva (HUG), Geneva, Switzerland; Department of Thoracic Surgery, Lausanne University Hospital (CHUV), Lausanne, Switzerland; Faculty of Biology and Medicine, University of Lausanne (UNIL), Lausanne, Switzerland; Department of Thoracic Surgery, Lausanne University Hospital (CHUV), Lausanne, Switzerland; Faculty of Biology and Medicine, University of Lausanne (UNIL), Lausanne, Switzerland

**Keywords:** Video-assisted thoracoscopic surgery, Segmentectomy, Lobectomy, Non-small-cell lung carcinoma, Survival

## Abstract

**OBJECTIVES:**

The aim of this study was to compare short-term outcomes and local control in pT1c pN0 non-small-cell lung cancer that were intentionally treated by video-assisted thoracoscopic surgery (VATS) lobectomy or segmentectomy.

**METHODS:**

Multicentre retrospective study of consecutive patients undergoing VATS lobectomy (VL) or VATS segmentectomy (VS) for pT1c pN0 non-small-cell lung cancer from January 2014 to October 2021. Patients’ characteristics, postoperative outcomes and survival were compared.

**RESULTS:**

In total, 162 patients underwent VL (*n* = 81) or VS (*n* = 81). Except for age [median (interquartile range) 68 (60–73) vs 71 (65–76) years; *P* = 0.034] and past medical history of cancer (32% vs 48%; *P* = 0.038), there was no difference between VL and VS in terms of demographics and comorbidities. Overall 30-day postoperative morbidity was similar in both groups (34% vs 30%; *P* = 0.5). The median time for chest tube removal [3 (1–5) vs 2 (1–3) days; *P* = 0.002] and median postoperative length of stay [6 (4–9) vs 5 (3–7) days; *P* = 0.039] were in favour of the VS group. Significantly larger tumour size (mean ± standard deviation 25.1 ± 3.1 vs 23.6 ± 3.1 mm; *P* = 0.001) and an increased number of lymph nodes removal [median (interquartile range) 14 (9–23) vs 10 (6–15); *P* < 0.001] were found in the VL group. During the follow-up [median (interquartile range) 31 (14–48) months], no statistical difference was found for local and distant recurrence in VL groups (12.3%) and VS group (6.1%) (*P* = 0.183). Overall survival (80% vs 80%) was comparable between both groups (*P* = 0.166).

**CONCLUSIONS:**

Despite a short follow-up, our preliminary data shows that local control is comparable for VL and VS.

**Figure:**
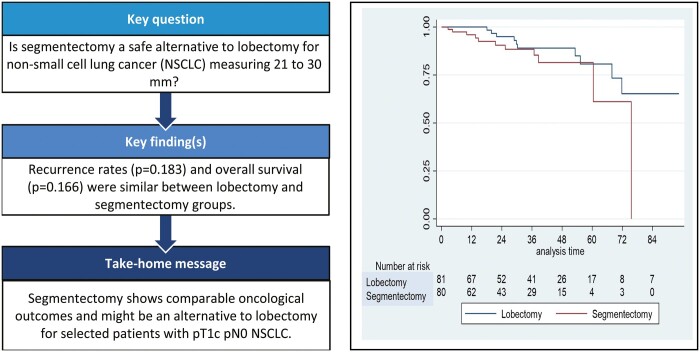

## INTRODUCTION

Lung cancer is a major cause of cancer-related death worldwide with surgical resection remaining the gold standard of treatment for early-stage tumours. To this day, lobectomy with mediastinal lymph node dissection is considered the gold standard procedure [1, 2]. Sublobar resections have long been proposed as an alternative treatment for patients deemed ineligible for lobectomy due to compromised lung function or major comorbidities. But Ginsberg’s first randomized trial, comparing lobar to sublobar approach revealed higher death rate and increased risk for local relapse when segmentectomies were performed [1].

However, given the rise of computed tomography (CT) screening programmes and improvement in diagnostic modality (thin-section CT), early detection, sizing and mapping of small size nodules has emerged. Consequently, the applied indications and opportunities of sublobar resections have been readdressed with more recent studies showing promising results for well-selected patients [3, 4].

Moreover, a recent randomized controlled trial (JCOG0802/WJOG4607L) indicated that segmentectomy could achieve identical recurrence and survival rates to lobectomy for early-stage (pT1 pN0 M0) non-small-cell lung cancer (NSCLC) ≤2 cm, as long as adequate surgical margins and systematic lymph node dissection are performed [5]. Likewise, controversy remains for tumours of larger size (>2 cm) [6–8]. Two retrospective propensity score-matched study including NSCLC of 2–3 cm found no difference in terms of overall survival (OS) and recurrence-free survival between patients benefiting lobectomy when compared to segmentectomy [6, 7]. On the other hand, Yu *et al.* [8] revealed a significantly worse survival in patients undergoing segmentectomy. Thus, the balance of preserving lung function by performing a parenchyma-sparing segmental resection for NSCLC >2 cm with the potential negative impact on survival and recurrence remains unclear.

The aim of this study was to compare short-term oncological outcomes (survival, local control) in patients with pT1c pN0 NSCLC that were intentionally treated by video-assisted thoracoscopic surgery (VATS) segmentectomy or lobectomy.

## PATIENTS AND METHODS

### Ethical statement

This study was approved by the Local Ethics Committee (CER-VD) and individual consent was waived (N°2022–01148).

### Study design and patient selection

We conducted a retrospective multicentre observational study reviewing all consecutive patients with pT1c pN0 NSCLC who underwent a planned complete (R0) VATS segmentectomy (VS) or VATS lobectomy (VL) with lymphadenectomy between January 2014 and October 2021. Patients were treated by 1 of the 5 board-certified thoracic surgeons in 4 different centres across Switzerland. All surgeons had a large experience in VATS anatomical resections. The study population included patients aged over 18 years who underwent VS or VL with mediastinal lymphadenectomy for pT1c pN0 NSCLC (adenocarcinoma, squamous cell carcinoma, large cell carcinoma) only. Eligible patients had to have no history of ipsilateral thoracotomy, no previous chemotherapy or radiotherapy. All patients received contrast-enhanced thoracic CT and a fluorodeoxyglucose-positron emission tomography CT within 30 days prior to the surgery. All tumours had a consolidation-to-tumour ratio of 0.5 or more. Exclusion criteria encompassed all other types of anatomical or extra-anatomical lung resections (wedge, bilobectomy, sleeve lobectomy, pneumonectomy), middle lobectomy, open procedures, synchronous tumour, histology different than previously cited (carcinoid tumour, small-cell lung carcinoma, minimally invasive adenocarcinoma), an 8th edition TNM stage different than pT1c pN0 R0, pleural invasion, multiple lesions, nodal involvement or incomplete resections. Two groups were defined according to the extension of the resection: VL or VS.

### Data collection

Data were blindly collected from a multicentric electronic database. It included: patient demographics and comorbidities, preoperative pulmonary functions, American Society of Anaesthesiologists score, oncological characteristics including lesion histology, size and localization, number of dissected lymph nodes and TNM stage (8th edition), type of surgical procedure, postoperative mortality and morbidity, readmissions and reoperations, length of drainage, postoperative length of stay, date of death or last contact and recurrence. Postoperative morbidity was defined as any adverse event changing patient management and occurring during the 90-postoperative day period. Cardiac complications included arrhythmia, cardiac ischaemia and cardiac failure. Pulmonary complications included pneumonia, pneumothorax, haemothorax, pleural empyema, air leak (≥5 days), acute respiratory distress syndrome, subcutaneous emphysema and chylothorax. Loco-regional recurrence was defined as any recurrence in the ipsilateral lung, hilum or mediastinum without evidence of distant metastasis. Distant recurrence was defined as any contralateral lung, hilum or mediastinum or extra-thoracic metastatic disease. The OS was calculated from the time of surgery to either death or last follow-up.

The primary outcome was the OS and local and distant recurrence rates. Secondary outcomes were the postoperative outcomes (conversion thoracotomy rate, 90-postoperative day morbidity, 30-postoperative day mortality, lengths of drainage and hospital stay, readmission and reoperation rates).

### Surgical approach and workup

Before surgery, all cases were individually discussed in a multidisciplinary tumour board. Preoperative assessment included a chest CT and fluorodeoxyglucose-positron emission tomography scan with maximum standard uptake values and a transthoracic or bronchoscopic biopsy of the lesion to determine the histology when technically feasible. In case of suspected lymph node involvement on preoperative imaging, an endobronchial ultrasound fine-needle aspiration or mediastinoscopy was performed before surgery. The choice of treatment modality (lobectomy versus segmentectomy) was driven by unmeasured patient characteristics, but we usually favoured a segmentectomy in case of smaller, peripherally located lesions in a specific segment with an achievable surgical margin of ≥2 cm.

Patients were operated under general anaesthesia with single lung ventilation by double lumen intubation. A standardized 3-port anterior approach (utility incision in the 4th intercostal space, 1 incision for the 10 mm 30° thoracoscope in the 7th intercostal space anteriorly and a 3rd incision posteriorly) or 1-port approach (since 2018) were used. Segmentectomy procedures were performed with individual dissection of the segmental bronchus, arteries and veins followed by a systematic hilar and mediastinal lymph node resection. Intersegmental plane was identified using systemic injection of indocyanine green when necessary and the dissection itself was performed by using stapling or energy device. Surgical margins were systematically evaluated and all types of segmentectomies were performed. In case of suspected hilar nodal involvement, a frozen section was performed and a completion lobectomy was undertaken if the nodal status was upstaged (N1). Surgical specimens were extracted through a protective bag.

All cases were discussed again after surgery in a multidisciplinary tumour board to assess the need of adjuvant chemotherapy. The follow-up consisted in chest CT scans every 3 months for the 1st 2 years, then every 6 months for a total of 5 years.

### Statistical analysis

Results are presented as numbers with percentages for binary variables, means with standard deviation for normally distributed continuous variables or medians with interquartile range for non-normally distributed continuous variables or nominal variables with large number categories. The following list of variables were considered not-normally distributed: age, number of dissected lymph node, operative time, length of drainage, postoperative length of stay and follow-up time. Numerical variables were compared between VL and VS groups using the unpaired Student’s *t*-test or the Mann–Whitney *U*-test according to the distribution. Categorical variables were compared using the Chi-squared test. OS was calculated using the Kaplan–Meier formula and compared with a log-rank test. A *P*-value < 0.05 was defined as the threshold for statistical significance. No inferential analysis was performed on the local versus distant recurrence due to the small sample size. The statistics provided are descriptive in nature. All statistical analyses were performed using the Stata version 14 software (StataCorp, College Station, TX, USA).

## RESULTS

A total of 162 patients with pT1c pN0 NSCLC underwent VL (*n* = 81, 50%) or VS (*n* = 81, 50%) for adenocarcinoma (*n* = 125, 77.1%), squamous cell carcinoma (*n* = 35, 21.6%) or large cell tumours (*n* = 2, 1.2%) (Table 1). Except an older age [median (interquartile range) 71 (65–76) vs 68 (60–73) years; *P* = 0.037] and a higher rate of previous cancer history (48.1% vs 32%; *P* = 0.038) observed in the VS group, patient characteristics were similar between both groups. NSCLC were slightly smaller in the VS group (mean ± standard deviation: 23.6 ± 3.1 vs 25.1 mm ± 3.1; *P* = 0.001) but there was no significant difference in histology subtype between groups. Most of the procedures concerned upper lobes (*n* = 109, 67.3%). All types of segmentectomy were performed as demonstrated in Table 2. The median number of dissected lymph nodes was lower in the VS group [10 (6–15) vs 14 (19–23); *P* < 0.001].

**Table 1:** Patient characteristics

Variables	Total (*n* = 162)	Lobectomy (*n* = 81)	Segmentectomy (*n* = 81)	*P*-Value
Sex (female), *n* (%)	79 (48.7)	39 (48.1)	40 (49.3)	0.875
Age (years), median (IQR)]	69 (63–74)	68 (60–73)	71 (65–76)	0.037
BMI (kg/m^2^), mean ± SD	25.3 ± 4.3	25.2 ± 4.3	25.9 ± 4.3	0.814
Comorbidities, *n* (%)				
Cardiopathy	46 (28.4)	20 (24.7)	26 (32)	0.297
High blood pressure	86 (53)	43 (53)	43 (53)	1
Atrial fibrillation	18 (11)	9 (11)	9 (11)	1
Tobacco	132 (81.4)	63 (77.7)	69 (85)	0.228
Diabetes	23 (14.1)	9 (11)	14 (17.2)	0.264
Kidney failure	16 (9.8)	7 (8.6)	9 (11)	0.599
Previous cancer	65 (40.1)	26 (32)	39 (48.1)	0.038
Preoperative pulmonary function, mean ± SD				
FEV1 (%)	86.03 ± 21.6	88.9 ± 21.1	86.1 ± 21.6	0.085
DLCO (%)	73.4 ± 22.4	75.8 ± 22.6	71.1 ± 22.3	0.194
ASA score	2.52 ± 0.54	2.53 ± 0.54	2.52 ± 0.54	0.329
Histology, *n* (%)				
Adenocarcinoma	125 (77.1)	60 (74)	65 (80)	0.351^a^
Squamous cell carcinoma	35 (21.6)	19 (23.4)	16 (19.8)	
Large cell tumour	2 (1)	2 (2)	0	
Size (mm), mean ± SD	24.3 ± 3.1	25.1 ± 3.1	23.6 ± 3.1	0.001
21–25, *n* (%)	114 (70.3)	46 (56.7)	68 (83.9)	<0.001^a^
25–30, *n* (%)	48 (29.7)	35 (43.3)	13 (16)	
Localization, *n* (%)				
Right upper lobe	57 (35.2)	33 (40.7)	24 (29.6)	0.186^a^
Right lower lobe	27 (16.7)	16 (19.8)	11 (13.6)	
Left upper lobe	52 (32.1)	21 (25.9)	31 (38.3)	
Left lower lobe	26 (16)	11 (13.6)	15 (18.5)	
Dissected lymph nodes (*n*), median (IQR)	12 (7–19)	14 (9–23)	10 (6–15)	<0.001
Operative time (min), median (IQR)	130 (103–157)	131 (105–162)	127 (100–155)	0.432
Adjuvant chemotherapy, *n* (%)	15 (9.2)	10 (12)	5 (6.1)	0.183

a Fisher’s exact test.

ASA: American Society of Anaesthesiologists; BMI: body mass index; DLCO: diffusing capacity of the lung for carbon monoxide; FEV1: forced expiratory volume in 1 s; IQR: interquartile range; SD: standard deviation.

**Table 2:** Video-assisted thoracoscopic surgery segmentectomy group

	Total (*n* = 81)	Right side (*n* = 35)	Left side (*n* = 46)
Single segmentectomy, *n* (%)	37 (45.7)	19	18
Multiple segmentectomy, *n* (%)	44 (54.3)	16	28
Simple segmentectomy, *n* (%)	36 (44.4)		
S1 + 2 + 3	16	0	16
S4 + 5	5	0	5
S6	13	5	8
S7 + 8 + 9 + 10	2	1	1
Complex segmentectomy, *n* (%)	45 (55.6)		
S1	11	6	5
S2	4	3	1
S3	4	2	2
S1 + 2	16	10	6
S1 + 3	2	2	0
S2 + 6	1	1	0
S8	4	2	2
S10	1	1	0
S9 + 10	2	2	0

Regarding the postoperative outcomes, the rate of conversion thoracotomy was similar between both groups (4.9% vs 4.9%; *P* = 1) (Table 3). These procedures were performed to control intraoperative bleeding (*n* = 3), in case of fused fissure (*n* = 2), pleural adhesions (*n* = 2) or anatomical variation (*n* = 1). The overall 90-postoperative day morbidity was similar between VL and VS groups (34.5% vs 29.6%; *P* = 0.501). One patient of the VS group (1.2%) died during the 30-postoperative day period because of acute respiratory distress syndrome. The rate of 30-day postoperative readmissions was statistically similar between both groups (1.2% vs 2.5%; *P* = 0.568). One patient in the VL group presented an inflammatory pleural effusion and 2 patients in the VS group presented a pneumothorax or a pleural empyema. All these complications were treated conservatively with pleural drainage and medical therapy. Three patients were re-operated during the 30-postoperative day period (1.9%). One patient in the VS presented a subcutaneous emphysema, which required a surgical exploration, decortication and pleural drainage. The other 2 patients, 1 in each group, underwent clot removal and pleural drainage for haemothorax. Both the median length of drainage [2 days (1–3) vs 3 days (1–5); *P* = 0.002] and the median postoperative length of stay [5 days (3–7) vs 6 days (4–9); *P* = 0.039) were reduced by 1 day in the VS group.

**Table 3:** Postoperative outcomes after video-assisted thoracoscopic surgery lobectomy or video-assisted thoracoscopic surgery segmentectomy

Variables	Total (*n* = 162)	Lobectomy (*n* = 81)	Segmentectomy (*n* = 81)	*P*-Value
Conversion thoracotomy, *n* (%)	8 (4.9)	4 (4.9)	4 (4.9)	1
Mortality (30 days), *n* (%)	1 (0.6)	0	1 (1.2)	0.937
Overall morbidity (90 days), *n* (%)	52 (32)	28 (34.5)	24 (29.6)	0.501
Pulmonary complications	42 (25.9)	23 (28.4)	19 (23.4)	0.474
Cardiac complications	12 (7.4)	5 (6.1)	7 (8.6)	0.550
Re-operation, *n* (%)	3 (1.9)	1 (1.2)	2 (2.5)	0.568
Re-admission, *n* (%)	3 (1.9)	1 (1.2)	2 (2.5)	0.568
Length of drainage (days), median (IQR)	2 (1–4)	3 (1–5)	2 (1–3)	0.002
Postoperative length of stay (days), median (IQR)	5 (3–8)	6 (4–9)	5 (3–7)	0.039
Follow-up (months), median (IQR)]	31 (14–48)	32 (14–49)	30 (14–48)	0.009
Follow-up index	0.91	0.91	0.90	
Overall recurrence, *n* (%)	15 (9.2)	10 (12.3)	5 (6.1)	0.183
Local recurrence only	6 (3.8)	4 (4.9)	2 (2.4)	0.414
Mediastinal lymph node	6 (3.8)	4 (4.9)	2 (2.4)	0.414
Ipsilateral lung	0	0	0	
Local and distant	1 (0.6)	1 (1.2)	0	
Distant	7 (4.3)	4 (4.9)	3 (3.7)	0.700
New lung cancer, *n* (%)	5 (3.1)	3 (3.7)	2 (2.5)	0.901
Other cancer, *n* (%)	13 (8)	5 (6.2)	8 (9.9)	0.796
Death-specific lung cancer, *n* (%)	10 (6.2)	7 (8.6)	3 (3.7)	0.733

IQR: interquartile range.

**Figure 1:**
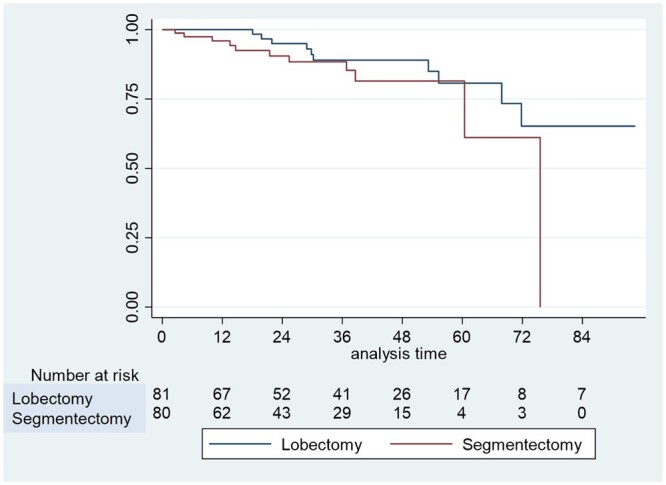
Kaplan–Meier curve of overall survival after video-assisted thoracoscopic surgery segmentectomy and video-assisted thoracoscopic surgery lobectomy in patients with pT1c pN0 R0 non-small-cell lung cancer.

During the median follow-up of 31 months (14–48 months), 10 patients (12.3%) in the VL group and 5 patients (6.1%) in the VS group presented recurrence (*P* = 0.183) (Table 3). Concerning local recurrence, we did not observe any recurrence in the ipsilateral lung. The 5-year OS rate was similar between both groups (80% vs 80%; *P* = 0.166) (Fig. 1).

## DISCUSSION

Here, we present 162 patients with pT1c pN0 NSCLC undergoing complete resection by either VL or VS with systemic hilar and mediastinal lymph node dissection. We did not find any difference in terms of postoperative outcomes at 30 days or oncological control over the median follow-up period of 31 months, whether this was loco-regional or distant relapses. Finally, the OS was similar in both groups.

Since many years, lobectomy with lymph node dissection remained the standard treatment of early-stage NSCLC [2]. Sublobar anatomical resections (segmentectomy) were exclusively proposed to patients with several comorbidities or decreased pulmonary function. However, with the development of minimally invasive surgical approach and thin-slice CT scans or 3D reconstruction allowing a precise description of bronchovascular segmental and tumoural anatomy, segmentectomy became technically easier to perform. Moreover, it has been shown that postoperative morbidity rates were similar between segmentectomy and lobectomy [9, 10], as demonstrated in our study. Thus, segmentectomy has gained popularity to treat early-stage NSCLC with oncological outcomes showing promising results. Lately, the database analysis of the Society of Thoracic Surgeons demonstrated a similar OS rate after segmentectomy when compared to lobectomy for NSCLC in clinical stage IA (hazard ratio 1.04; *P* = 0.64) [11]. We also previously reported on 188 patients with NSCLC <2 cm who underwent VS or VL with a median follow-up of 23 months, finding similar ipsilateral pulmonary (*P* = 0.388) or distant (*P* = 0.124) recurrence rates and OSs (93% vs 92%; *P* = 0.738) [3]. A recent meta-analysis by Winckelmans *et al.* [12] including 28 studies corroborated these results by demonstrating a significantly decreased OS and recurrence-free survival after segmentectomy for stage IA NSCLC. Another large study performing a cox regression analysis on 774 propensity-matched patients and another one with a longer follow-up corroborated these findings [13, 14]. However, the recurrent retrospective design allowed for some selective bias. Recently, a randomized controlled trial comparing segmentectomy and lobectomy for NSCLC of ≤2 cm was published [5]. This trial demonstrated a significantly improved OS after segmentectomy and a similar 5-year relapse-free survival, despite an increased local recurrence rate after segmentectomy [5]. Thus, sublobar resections seem to be an appropriate alternative to lobectomy in this cancer population. Another prospective randomized controlled trial evaluating the same population is ongoing and will provide more results that could potentially change the standards for this specific population [15].

Regarding sublobar resections for NSCLC of larger size (>2 cm), data are still a subject of controversy. Indeed, tumour size is an important prognostic factor of survival for NSCLC as demonstrated by Cao *et al.* [16]. Their retrospective propensity-matched study reviewed 16 819 stage IA NSCLC (8th edition) who underwent surgical resection and found a similar survival after lobectomy or segmentectomy for tumours from 11 and 20 mm but a better survival after lobectomy for tumours from 21 to 30 mm [16]. The Surveillance, Epidemiology and End Results database in the USA provided similar conclusions with a decreased survival rate after segmentectomy for NSCLC of >2–5 cm when compare to lobectomy [17]. This study emphasized the tumour size effect on mortality by doing a size stratification in statistical analysis which showed that for each 1-cm size increment, the risk of death was increased in segmentectomy group [17]. Finally, other studies that were focused on T1c NSCLC only had some controversial results [6–8]. Indeed, 2 studies did a retrospective propensity-matched analysis showing similar OS and recurrence-free survival when comparing both groups for the treatment of T1c NSCLC with a median follow-up of 4–5 years [6, 7]. On the other hand, Surveillance, Epidemiology and End Results database revealed a decreased OS after segmentectomy (hazard ratio 1.348; *P* < 0.001) for NSCLC measuring 21–30 mm [8]. In this study, the follow-up was quite short (median 48 months) and it is to note that the number of resected lymph node was quite low in the segmentectomy group (median 4.4), which might have had an influence on the measured difference in OS [8]. However, these studies included open procedures (thoracotomy) and heterogeneous populations. In our study, we included a specific population of patients with pT1c pN0 R0 NSCLC undergoing VATS procedures. Even though we did not analyse a single histological entity, we selected only the 3 most commonly encountered histologic subtypes (adenocarcinoma, squamous cell carcinoma, large cell carcinoma). Our results support the concept that segmentectomy shows comparable oncological outcomes (local control, survival) to lobectomy even though our follow-up was quite short as compared to the study of Chan *et al.* However, it is to note that we observed a significant difference in tumour size between both groups (*P* = 0.001), with smaller tumours in VS group. Although we did not perform a propensity score matching, which limits the interpretation of our conclusions, we observed that most of patients’ characteristics were statistically similar and we can thus consider that VS seems to be safe and efficient for small, peripherally located lesions.

Yet, sublobar resections remain technically challenging procedures. In our study, we performed all types of segmentectomy on both sides and included quadri-segmentectomy of the lower lobes in the VS group as other previous studies [6, 8]. VS are not always feasible. Patients with past thoracic procedures could potentially show more difficult bronchovascular dissections and less identifiable anatomical structures. When compared to a simple lobectomy, tumour localization and its predicted size are more to be considered during segmentectomy. Smaller tumours, peripherally located in specific segments, are more easily resectable with sufficient safety margins. This might be an explanation on why we found to perform more VS in older patients (*P* = 0.037) with smaller tumours (*P* = 0.001) and fewer dissected lymph nodes (*P* < 0.001), although none of these observations was deemed completely unexpected. It is noteworthy that none was observed to be associated with higher rates of local recurrences in this group of patients. Another interesting difference observed in our study was that in the VS group, patients presented a higher rate of previous cancer history (*P* = 0.038), thus making them probably more fragile and influencing our choice of parenchyma-sparing strategies. This trend was also described by other groups [6, 8, 11].

A systematic hilar and mediastinal nodal dissection was also performed in all of our cases, in view of a known increased risk of nodal metastasis in NSCLC ≥2 cm [18]. Although our results demonstrated a significantly lower number of dissected lymph nodes in the VS group (*P* < 0.001), the mediastinal lymph node recurrence rate was similar between VL and VS groups (*P* = 0.414). Other studies also found significantly fewer dissected lymph nodes in the segmentectomy groups [6–8]. This might be explained by the easier access to interlobar and hilar nodal stations when performing lobectomy as compared to segmentectomy procedures. The number of dissected lymph node has been identified as a strong predictor of survival in early-stage NSCLC [19–21] and a threshold of at least 5 resected lymph nodes for lobectomy procedures was recently mentioned by Dezube *et al.* [19], number which was achieved in all of our cases.

In our study, anatomical resections were all performed with at least 2 cm of safety margins, a standard threshold that is now accepted [22]. This might have contributed to the ‘no recurrence’ in ipsilateral lungs. Our overall recurrence rate of 9.2% was similar to the values described in other centres [7].

### Limitations

Our study presents several limitations, the first one being its retrospective design allowing missing information and the small sample size with inherent heterogeneity due to the participation of 5 different centres potentially limiting the statistical significance of our results. Secondly, our short-term follow-up period limits the drawing of conclusion about our survival and oncological control results. Then, missing data about the choice of performing 1 surgical approach rather than another (lobectomy versus segmentectomy) might have induced a selection bias. Finally, we did not perform a propensity-matched analysis, limiting interpretation of our results. It is thus impossible to rule out that the observed differences are due to factors other than the surgical technique.

## CONCLUSION

In conclusion, despite a short follow-up period, our preliminary data shows that local control is comparable between VL and VS for pT1c pN0 NSCLC. Further prospective randomized trials are needed to corroborate these results.

## Data Availability

The data underlying this article will be shared on reasonable request to the corresponding author.

## Notes

Presented at the annual ESTS meeting in the Hague (Netherland) during the pulmonary neoplastic I—(early-stage lung cancer) Session V, 20 June 2022.

## ABBREVIATIONS

CT: Computed tomography
NSCLC: Non-small-cell lung cancer
OS: Overall survival
VATS: Video-assisted thoracoscopic surgery
VL: Video-assisted thoracoscopic surgery lobectomy
VS: Video-assisted thoracoscopic surgery segmentectomy
